# Translation and validation into Spanish of the Technology Acceptance Model (TAM-12) in adults: a structural equation modeling and Bayesian reliability analysis

**DOI:** 10.3389/fpsyg.2026.1864863

**Published:** 2026-07-31

**Authors:** Delly Santos-Chuquispuma, Renzo Santos-Chuquispuma, Jonatan Baños-Chaparro

**Affiliations:** Programa Académico de Psicología, Facultad de Ciencias de la Salud, Universidad Privada Norbert Wiener, Lima, Peru

**Keywords:** adults, Bayesian reliability, confirmatory factor analysis, psychometrics, technology acceptance model

## Abstract

**Introduction:**

Digital transformation has established technology acceptance as a key factor for productivity and well-being in workplace environments. However, psychometric evidence regarding the Technology Acceptance Model (TAM-12) in Spanish-speaking adult populations remains heterogeneous, and there are no validated adaptations for the Peruvian context.

**Objective:**

To analyze the psychometric properties of TAM-12 in adult workers from the private technology sector in Metropolitan Lima.

**Methods:**

A total of 496 adult workers from the private technology sector participated, completing a sociodemographic questionnaire, the TAM-12, and the Resistance to Change Scale (RCT-10). For statistical analysis, structural equation modeling with a robust maximum likelihood estimator and Bayesian reliability was applied.

**Results:**

TAM-12 demonstrated adequate content validity (V > 0.70), a two-dimensional structure (CFI = 0.92, RMSEA = 0.07 [90% CI: 0.067, 0.083], SRMR = 0.05), and high reliability for the perceived usefulness dimension (*ω* = 0.92 [95% CI: 0.91, 0.93], H = 0.93) and for perceived ease of use (ω = 0.90 [95% CI: 0.88, 0.92], H = 0.90). Additionally, both dimensions showed negative and statistically significant associations with resistance to change (*r* = −0.57 and *r* = −0.44, respectively).

**Conclusion:**

TAM-12 showed initial evidence of adequate psychometric performance for interpreting its scores in adult workers from the Peruvian private technology sector. However, further studies are needed to examine measurement invariance, convergent validity, discriminant validity, and temporal stability.

## Introduction

Digital transformation has profoundly reshaped work environments on a global scale, positioning the adoption of intelligent technologies as a determining factor for organizational competitiveness and workers’ well-being. In this context, the Technology Acceptance Model (TAM), proposed by Davis (1989), remains one of the most robust theoretical frameworks for understanding and predicting individual behavior toward digital innovation. TAM is fundamentally based on two constructs: perceived usefulness—understood as the belief that technology will enhance job performance—and perceived ease of use—referring to the expectation that its use will not require considerable effort (Davis, 1989). The strength of this model has been empirically validated in various studies and organizational contexts; a recent meta-analysis highlighted its robustness as a predictor of intention to use and actual use of technology in workplace environments (Marikyan et al., 2023). However, although digitalization has become a strategic axis for productivity in Latin America, academic literature on moderating factors in the region remains limited (Ciarli et al., 2021; Cordero et al., 2023; Vélez and Morero, 2024). In Peru, particularly in Metropolitan Lima—where approximately 70% of national technology companies are concentrated (United Nations Conference on Trade and Development [UNCTAD], 2023)—the technological acceleration driven by private investment and international organizations (Inter-American Development Bank (IDB), 2023; Espina-Romero et al., 2024) positions workers in this sector as a priority unit of analysis for understanding processes of technological appropriation in the South American context.

Workers in the private technology sector operate in an environment that requires continuous adaptation to constantly evolving digital tools, exposing them systematically to the psychosocial challenges inherent in digital transformation (Cirillo et al., 2023; Hilda et al., 2024). Among the factors that modulate this adaptation, resistance to change occupies a central role: it is a complex phenomenon reflecting employees’ reluctance to adopt innovations, shaped by psychological, cultural, and organizational dynamics (Scholkmann, 2021). From the TAM perspective, resistance to change can act as a barrier that reduces both perceived usefulness and perceived ease of use of technological innovations, thereby interfering with the intention to adopt them (Green, 2024; Seppänen et al., 2025). Previous research has shown that concerns associated with resistance to change—such as fear of job loss or low technological self-efficacy—are relevant predictors of opposition to adopting new tools (Rahman et al., 2024), and that such resistance may decrease when workers have adequate emotional and organizational resources (Van der Westhuizen, 2021). In this sense, studying the relationship between technology adoption and resistance to change is essential to understanding the psychosocial dynamics that facilitate or hinder technological integration in the Peruvian workplace.

To operationalize the core constructs of TAM within the framework of this study, the TAM-12 will be used, a twelve-item version that assesses the dimensions of perceived usefulness and perceived ease of use through a seven-point Likert scale, with reported reliability indices of up to *α* = 0.98 in workplace contexts (Venkatesh and Davis, 2000). TAM-12 has been applied in multiple cultural and work contexts worldwide, supporting its versatility and cross-cultural validity. A review of the international literature shows robust psychometric evidence across various countries and languages, including the United States, China, Spain, Mexico, Chile, and Colombia (DeLange Martinez et al., 2025; De la Peña-López and Acosta-Gonzaga, 2025; Grandón et al., 2021; Pino Varela, 2022). Specifically, in Spanish-speaking contexts, adaptations into Spanish have demonstrated that the instrument maintains its bifactorial structure and high internal consistency, with Cronbach’s alpha coefficients above.85 (Navarro et al., 2023; Silvestre et al., 2022). Recent systematic reviews further suggest that TAM-12 is one of the most robust instruments for contemporary organizational research due to its flexibility in understanding user interaction across various sociotechnical systems, from e-commerce to artificial intelligence (Yurder, 2025). However, most of these validations have been conducted with samples of teachers and university students (Lobos et al., 2022; Grandón et al., 2021) or general adult populations, leaving a knowledge gap regarding its psychometric performance among workers in the private technology sector in Latin American contexts. To examine validity based on relationships with other variables, the Resistance to Change Scale (RCT-10), adapted to the Peruvian context by Luna-Victoria and Guevara (2021) with adequate psychometric properties, was also used, allowing the performance of TAM-12 to be linked with the study’s central psychosocial variable.

Although these Spanish-language validations constitute important antecedents, their direct transfer to the Peruvian organizational context cannot be assumed. Measurement instruments adapted in one Spanish-speaking country may preserve linguistic equivalence but still differ in terms of occupational vocabulary, digitalization patterns, organizational culture, and workers’ exposure to intelligent technologies. In addition, previous Spanish-language validations have mostly focused on educational or general user populations rather than workers from the private technology sector. Therefore, a Peruvian validation is not justified merely by the absence of a local version, but by the need to examine whether the two-dimensional structure, reliability, and external associations of TAM-12 remain stable in a specific organizational setting characterized by accelerated technological change.

The available psychometric literature on TAM-12 presents additional limitations that justify the present study. Most existing research has not incorporated Bayesian approaches to reliability estimation, which provide more stable estimates with credibility intervals, especially in moderate sample sizes (Pfadt et al., 2023). Likewise, few studies have employed structural equation modeling in Latin American contexts to analyze the relationship between TAM-12 dimensions and psychosocial variables (Silvestre et al., 2022; Navarro et al., 2023). More importantly for the purposes of this study, there is no evidence of adaptation or validation of TAM-12 into Spanish for the Peruvian adult population, representing a significant limitation for organizational research in the country and a clear opportunity for empirical contribution.

Beyond the existence of prior validations, the available evidence shows several unresolved issues. First, much of the Spanish-language literature has emphasized internal consistency and factor replication without sufficiently examining the behavior of TAM-12 in organizational samples exposed to continuous technological change (De la Peña-López and Acosta-Gonzaga, 2025; Grandón et al., 2021; Pino Varela, 2022). Second, previous studies have commonly relied on classical reliability indicators, mainly Cronbach’s alpha, which may be limited when tau-equivalence is not met (Pfadt et al., 2023). Third, fewer studies have connected TAM-12 scores with psychosocial variables that may operate as barriers to technology adoption, such as resistance to change (Oreg, 2003). Consequently, the present study seeks to provide incremental psychometric evidence by combining confirmatory factor analysis, Bayesian omega, coefficient H, and validity evidence based on relationships with resistance to change in Peruvian technology-sector workers.

Based on this background, the general aim of this research is to examine the psychometric properties of TAM-12 in adults from the private technology sector in Metropolitan Lima. Specifically, the study aims to: (a) evaluate the content validity of the instrument; (b) analyze its factorial structure through confirmatory factor analysis; (c) estimate the reliability of TAM-12 using Bayesian omega and coefficient H; and (d) examine the relationship between TAM-12 and resistance to change as evidence of validity based on relationships with other variables. These analyses will allow determining the suitability of TAM-12 as a valid and reliable tool for assessing technology adoption in Peruvian adults, contributing to the advancement of research in organizational psychology and to the design of strategies that promote more effective technological integration in the private sector.

## Methods

### Design

The study corresponds to an instrumental design, in which the psychometric properties of TAM-12 are analyzed (Ato et al., 2013). Additionally, it is a basic, cross-sectional study with a quantitative approach, aimed at examining the validity and reliability of the instrument in a sample of adults.

### Participants

A total of 496 workers from the private technology sector in Metropolitan Lima participated in the study. Participants were selected through non-probability convenience sampling and met the following inclusion criteria: (a) being 18 years of age or older, (b) residing in Metropolitan Lima, (c) having at least 6 months of work experience in the private technology sector, and (d) using intelligent technologies in their daily work routine, such as automation software, artificial intelligence-based tools, or other advanced digital platforms. The exclusion criteria were: (a) presenting questionnaires with incomplete responses in the sociodemographic section or psychological instruments, and (b) voluntarily withdrawing from the survey before completing it. No missing data were identified; therefore, no imputation procedure was applied. The sample size estimation was based on the hypothetical factorial model of TAM-12 (two factors, 12 items) and considered four parameters: an expected CFI of 0.90, an average factor loading of 0.60, a statistical power of 99%, and a significance level of *p* = 0.05 (Arifin, 2026). The minimum required sample size was 257 participants. The final sample of the study exceeded this recommendation.

Table 1 presents the sociodemographic characteristics of the participants. The mean age was 30.99 years (SD = 8.46), ranging from 18 to 61 years. Regarding sex, the majority identified as male (53.0%), followed by female (47.0%). In terms of marital status, most participants were single (65.9%), followed by married (22.2%), cohabiting (8.7%), and divorced (2.8%), while 0.4% reported being widowed. Concerning educational level, the largest proportion reported completed university education (34.1%), followed by incomplete university education (28.4%) and completed postgraduate studies (16.5%). Smaller proportions included those with completed technical education (7.1%), incomplete technical education (1.4%), completed secondary education (2.0%), and completed primary education (0.2%). Regarding current job position, the most represented group consisted of full-time employees (34.1%), followed by self-employed workers or freelancers (17.7%), interns (16.1%), entrepreneurs or business owners (15.2%), and part-time employees (14.9%). In terms of work modality, the hybrid modality predominated (38.5%), followed by on-site work (32.1%) and remote work (29.4%). Finally, the largest proportion of participants reported more than 5 years of work experience (38.1%), followed by less than 1 year (25.8%), 2 to 5 years (22.2%), and between one and 2 years (13.9%). These results indicate a sample primarily composed of young adults with university education and diverse work experience within the technology sector.

**Table 1:** Sociodemographic characteristics of the participants.

Variables	*n* (%)
Sex	
Male	263 (53.0%)
Female	233 (47.0%)
Age	30.99 (DE = 8.46)
Marital status	
Single	327 (65.9%)
Married	110 (22.2%)
Cohabiting	43 (8.7%)
Divorced	14 (2.8%)
Widowed	2 (0.4%)
Education level	
Completed primary	1 (0.2%)
Completed secondary	10 (2.0%)
Incomplete technical	7 (1.4%)
Completed technical	35 (7.1%)
Incomplete university	140 (28.2%)
Completed university	169 (34.1%)
Incomplete postgraduate	52 (10.5%)
Completed postgraduate	82 (16.5%)
Current position	
Entrepreneur or business owner	75 (15.2%)
Self-employed or freelancer	88 (17.7%)
Part-time employee	74 (14.9%)
Full-time employee	179 (36.1%)
Intern	80 (16.1%)
Work modality	
On-site	159 (32.1%)
Remote	146 (29.4%)
Hybrid	191 (38.5%)
Work experience	
Less than 1 year	128 (25.8%)
1 to 2 years	69 (13.9%)
2 to 5 years	110 (22.2%)
More than 5 years	189 (38.1%)

### Instruments

#### Sociodemographic information

A section was included to collect participants’ sociodemographic data, including sex, age, marital status, educational level, current position within the technology sector, work modality, and years of work experience. These variables made it possible to characterize the sample and describe the profile of workers from the private technology sector who participated in the study.

#### Technology Acceptance Model (TAM-12)

This instrument consists of a 12-item questionnaire designed to assess the adoption of technologies in organizational contexts. The scale measures two main dimensions: perceived usefulness (items 1 to 6) and perceived ease of use (items 7 to 12). Item responses are measured using a 7-point Likert-type scale, ranging from 1 = extremely unlikely to 7 = extremely likely, where higher scores indicate a more favorable disposition toward technology (Davis, 1989). Previous studies in English have reported high levels of internal consistency (*α* = 0.98 and α = 0.94 for perceived usefulness and perceived ease of use, respectively) in organizational contexts (Venkatesh and Davis, 2000).

For the adaptation of the instrument into Spanish, the back-translation method was used. In the first stage, a professional bilingual translator with experience in psychological and organizational terminology independently translated the scale from English into Spanish. Subsequently, a second translator independently performed the back-translation from Spanish into English. After completing this process, both translators, together with the research team, conducted a joint review to reach consensus and validate the final version. During this phase, discrepancies between the original and back-translated versions were systematically examined, with special emphasis on semantic equivalence, conceptual coherence, and cultural appropriateness. Identified differences were resolved through discussion and agreement, prioritizing conceptual equivalence over literal translation.

Next, five expert judges in organizational and clinical psychology (with experience in psychometrics and occupational mental health) evaluated the items considering three fundamental criteria: relevance, representativeness, and clarity. They were also asked to assess the cultural relevance and contextual appropriateness of each item in relation to the Peruvian work environment. Finally, before the formal administration of the instrument, a pilot phase was conducted with a preliminary sample of ten adult individuals to assess the clarity and comprehension of the items in the target population. Participants were selected to represent different educational levels, and a brief cognitive probing was conducted to confirm proper understanding and interpretation of the items. No observations requiring modifications were recorded, suggesting adequate linguistic clarity and acceptance of the items in the study context. Table 2 presents both the original English version and its corresponding Spanish translation.

**Table 2:** Final Spanish version of the TAM-12.

English version	Spanish version
1. Using [this product] in my job would enable me to accomplish tasks more quickly	Utilizar [este producto] en mi trabajo me permitirá cumplir mis deberes de manera más rápida.
2. Using [this product] would improve my job performance.	Utilizar [este producto] mejoraría mi rendimiento laboral.
3. Using [this product] in my job would increase my productivity.	Utilizar [este producto] en mi trabajo incrementará mi productividad.
4. Using [this product] would enhance my effectiveness on the job.	Utilizar [este producto] mejoraría mi eficacia en el trabajo.
5. Using [this product] would make it easier to do my job.	Utilizar [este producto] me facilitaría hacer mi trabajo.
6. I would find [this product] useful in my job.	Consideraría útil [este producto] para mi trabajo.
7. Learning to operate [this product] would be easy for me.	Aprender a usar [este producto] me resultaría fácil.
8. I would find it easy to get [this product] to do what I want it to do.	Me resultaría fácil lograr que [este producto] haga lo que yo quiero.
9. My interaction with [this product] would be clear and understandable.	Mi interacción con [este producto] sería clara y comprensible.
10. I would find [this product] would be clear and understandable.	Consideraría que [este producto] es clara y comprensible.
11. It would be easy for me to become skillful at using [this product].	Sería fácil para mí adquirir habilidad en el uso de [este producto].
12. I would find [this product] easy to use.	Consideraría que [este producto] es fácil de usar.

#### Resistance to Change Scale (RTC-10)

This instrument assesses resistance to change in the workplace, specifically in relation to workers’ attitudes, emotions, and behaviors toward the introduction of intelligent technologies in the work environment (Oreg, 2003). The scale consists of 10 items, which are measured using a five-point Likert-type response format, ranging from 1 (Strongly disagree) to 5 (Strongly agree). The instrument has shown adequate levels of internal consistency, with an omega coefficient of 0.82, indicating satisfactory reliability for use in psychological research. In this study, a version adapted to the Peruvian context of Metropolitan Lima was used (Luna-Victoria and Guevara, 2021).

### Procedure

Data collection was carried out virtually through the implementation of an online questionnaire during the period between October and December 2025. The instrument was developed and hosted on the Google Forms platform, and its distribution was conducted through the researchers’ social media, which significantly expanded its reach to diverse profiles of potential participants. Prior to participation, respondents received comprehensive information about the study’s objectives, the anonymous nature of their participation, the strictly academic use of the collected data, and the procedures related to data handling. In addition, informed consent was requested as an essential requirement to proceed with the questionnaire.

The use of surveys in digital environments presents multiple methodological advantages. Among them are expanded access to the target population, the ability to systematically manage and monitor responses, flexibility in using various dissemination channels, and efficiency in terms of time and costs in the data collection process. These characteristics contribute to optimizing both available resources and the quality of the research process (Gosling and Mason, 2015).

### Data analysis

Statistical processing was carried out using RStudio software (version 4.3.2), following a sequential approach structured in different stages for the psychometric evaluation of TAM-12. In the first stage, a comprehensive descriptive analysis of the items was conducted to examine their statistical performance and determine their initial quality. For this purpose, indicators of central tendency and dispersion (mean and standard deviation) were calculated, as well as measures of distribution shape, specifically skewness and kurtosis. Additionally, a Pearson correlation matrix was constructed in accordance with the continuous nature of the items, along with corrected item–total correlations, establishing values above 0.30 as the minimum acceptance criterion (Kline, 2023). Content validity was also assessed using Aiken’s V coefficient, considering values above 0.70 as adequate, in line with existing methodological recommendations (Roebianto et al., 2023).

In the second stage, a confirmatory factor analysis (CFA) was conducted to examine the internal structure of the instrument. Although the items were measured using a 7-point Likert-type response format, they were treated as approximately continuous because response scales with five or more categories are commonly analyzed as continuous indicators in CFA, particularly when the aim is to evaluate the latent structure of psychometric instruments. Therefore, the robust maximum likelihood estimator (MLR) was used, as it is appropriate for this type of measurement and provides robust standard errors and a scaled chi-square statistic that corrects for deviations from multivariate normality (Kline, 2023). Although WLSMV is frequently recommended for ordinal indicators, MLR was selected because the seven response categories allowed the data to be treated as approximately continuous, while also accounting for the non-normal distribution observed in some items. Model fit quality was determined using various goodness-of-fit indices, including the Comparative Fit Index (CFI), the Root Mean Square Error of Approximation (RMSEA), and the Standardized Root Mean Square Residual (SRMR). Values above 0.90 for CFI and below 0.08 for RMSEA and SRMR were considered indicative of acceptable fit (Jordan Muiños, 2021). Additionally, modification indices (MI > 10) and standardized factor loadings were examined, with values above 0.30 considered acceptable (Kline, 2023).

In the third stage, the internal consistency of the instrument was evaluated using Bayesian omega (*ω*) and the H coefficient (Baños-Chaparro and Caycho-Rodríguez, 2024; Gunnell, 2020). The use of Bayesian omega was prioritized due to its ability to generate more robust estimates, including credibility intervals, especially in contexts with moderate sample sizes (Pfadt et al., 2023). Unlike traditional approaches, such as ordinal alpha or classical omega, Bayesian methods treat parameters as random variables and incorporate prior information, which helps increase estimation precision and reduce bias in finite samples (Pfadt et al., 2022). In this study, the prior distribution was set at *r* = 0.707, in accordance with simulation studies (Pfadt et al., 2023). In addition, model convergence was evaluated using the R-hat index, with values close to 1 indicating adequate convergence of the Markov chains, based on 1,000 iterations (Pfadt et al., 2022).

Finally, in the fourth stage, a covariance-based structural equation model (CB-SEM) was estimated to analyze the relationships between the latent variables of the study. For this purpose, the MLR estimator was used again, and overall model fit was evaluated using the CFI, RMSEA, and SRMR indices, following the same criteria established previously (Jordan Muiños, 2021). The magnitude of relationships was interpreted according to the cutoffs proposed by Cohen (1988), considering small effects = 0.10, moderate = 0.30, and large = 0.50.

### Ethical considerations

The study was conducted following the ethical guidelines of the American Psychological Association and the Code of Ethics of the Colegio de Psicólogos del Perú, as described in Chapter Six on good research practices (Colegio de Psicólogos del Perú, 2024; Knapp and VandeCreek, 2003). Prior to data collection, all participants were informed about the nature and objectives of the study and provided their informed consent electronically. Participation was entirely voluntary, the survey was administered anonymously, and rigorous measures were implemented to ensure confidentiality and the secure handling of all collected data, in accordance with established ethical guidelines (Hilbig et al., 2022).

Additionally, the research protocol underwent formal ethical review and received a favorable evaluation from the Ethics Committee of Universidad Privada Norbert Wiener under registration number A0019-2025-CIEIC-UPNW, on July 9, 2025, prior to the beginning of data collection. This approval confirmed that the research procedures met the ethical and institutional standards required for studies involving human participants.

## Results

### Evidence of content-based validity

Expert judges evaluated the content of the items according to the criteria of relevance, representativeness, and clarity, obtaining Aiken’s V values above 0.70 (Table 3). These results indicate adequate content validity and an acceptable level of agreement among the panel of expert judges regarding the appropriateness of the items. However, because the expert panel was small (*n* = 5), some confidence intervals were relatively wide, which should be considered when interpreting the strength of the content validity evidence. Therefore, these findings should be understood as preliminary and should be complemented in future studies with additional expert reviews involving larger and more diverse panels. All judges (*n* = 5) approved the final version of TAM-12, including its semantic and contextual suitability for the target population. Likewise, in the pilot sample (*n* = 10), no suggestions or modifications were made. Feedback obtained through cognitive probing indicated that the items were clearly understood and interpreted as intended, supporting their linguistic naturalness and comprehensibility in the target context.

**Table 3:** Content validity of TAM-12 items.

Ítems	Relevance (*n* = 5)		Representativeness (*n* = 5)		Clarity (*n* = 5)	
	V	CI 95%	V	CI 95%	V	CI 95%
1	0.73	0.52, 0.87	0.87	0.67, 0.95	0.93	0.75, 0.99
2	0.93	0.75, 0.99	0.73	0.52, 0.87	0.73	0.52, 0.87
3	0.87	0.67, 0.95	0.93	0.75, 0.99	0.87	0.67, 0.95
4	0.93	0.75, 0.99	0.73	0.52, 0.87	0.73	0.52, 0.87
5	0.73	0.52, 0.87	0.87	0.67, 0.95	0.93	0.75, 0.99
6	0.83	0.65, 0.93	0.83	0.65, 0.93	0.83	0.65, 0.93
7	0.93	0.75, 0.99	0.93	0.75, 0.99	0.93	0.75, 0.99
8	1.00	0.87, 1.00	1.00	0.87, 1.00	1.00	0.87, 1.00
9	0.87	0.67, 0.95	0.93	0.75, 0.99	0.87	0.67, 0.95
10	0.73	0.52, 0.87	0.87	0.67, 0.95	0.93	0.75, 0.99
11	0.93	0.75, 0.99	1.00	0.87, 1.00	1.00	0.87, 1.00
12	0.87	0.67, 0.95	0.93	0.75, 0.99	0.87	0.67, 0.09

V = Aiken’s V, CI = confidence intervals.

### Descriptive analysis

Table 4 shows that the highest arithmetic mean was observed in item 6 (M = 6.45), while the lowest corresponded to item 8 (M = 5.70). Regarding variability, the highest standard deviation was found in item 3 (SD = 0.83), and the lowest in items 9 and 10 (SD = 0.70). Skewness and kurtosis values indicated notable deviations from normality, with acceptable negative skewness values (*g*₁ ± 1.5), although kurtosis values were high (*g*₂ > 1.5) across all items. These high kurtosis values indicate leptokurtic distributions, suggesting that responses were highly concentrated around specific response categories, mainly toward the upper end of the scale. Therefore, the descriptive means should be interpreted with caution, as they may reflect a strong clustering of responses rather than a normally distributed pattern. Moreover, considering that all item means were above 5.70 on a 7-point response scale, a potential ceiling effect should be acknowledged. This may limit the discriminative ability of the instrument among participants with high levels of perceived usefulness and perceived ease of use, because their responses tended to cluster near the upper end of the scale. Additionally, all corrected item–total correlations were satisfactory, exceeding the 0.30 criterion. Likewise, the Pearson correlation matrix did not show evidence of multicollinearity (*r* > 0.90) among the items.

**Table 4:** Descriptive analysis of TAM-12 items.

Item	M	SD	g_1_	g_2_	*r_it_*
1	6.36	0.77	−1.67	5.73	0.70
2	6.20	0.82	−1.80	6.75	0.68
3	6.32	0.83	−1.77	5.49	0.76
4	6.12	0.79	−1.14	2.88	0.65
5	6.17	0.78	−1.01	1.53	0.68
6	6.45	0.76	−1.60	3.67	0.75
7	5.97	0.80	−1.30	3.58	0.65
8	5.70	0.78	−1.36	3.46	0.73
9	5.81	0.70	−1.57	6.69	0.69
10	5.82	0.70	−0.96	1.92	0.65
11	6.02	0.77	−1.19	3.50	0.67
12	5.78	0.79	−0.96	2.29	0.58

M, media; SD, standard deviation, g_1_ = skewness, g_2_ = kurtosis, r_it_ = corrected item test correlation.

### Evidence based on internal structure

**Figure 1:**
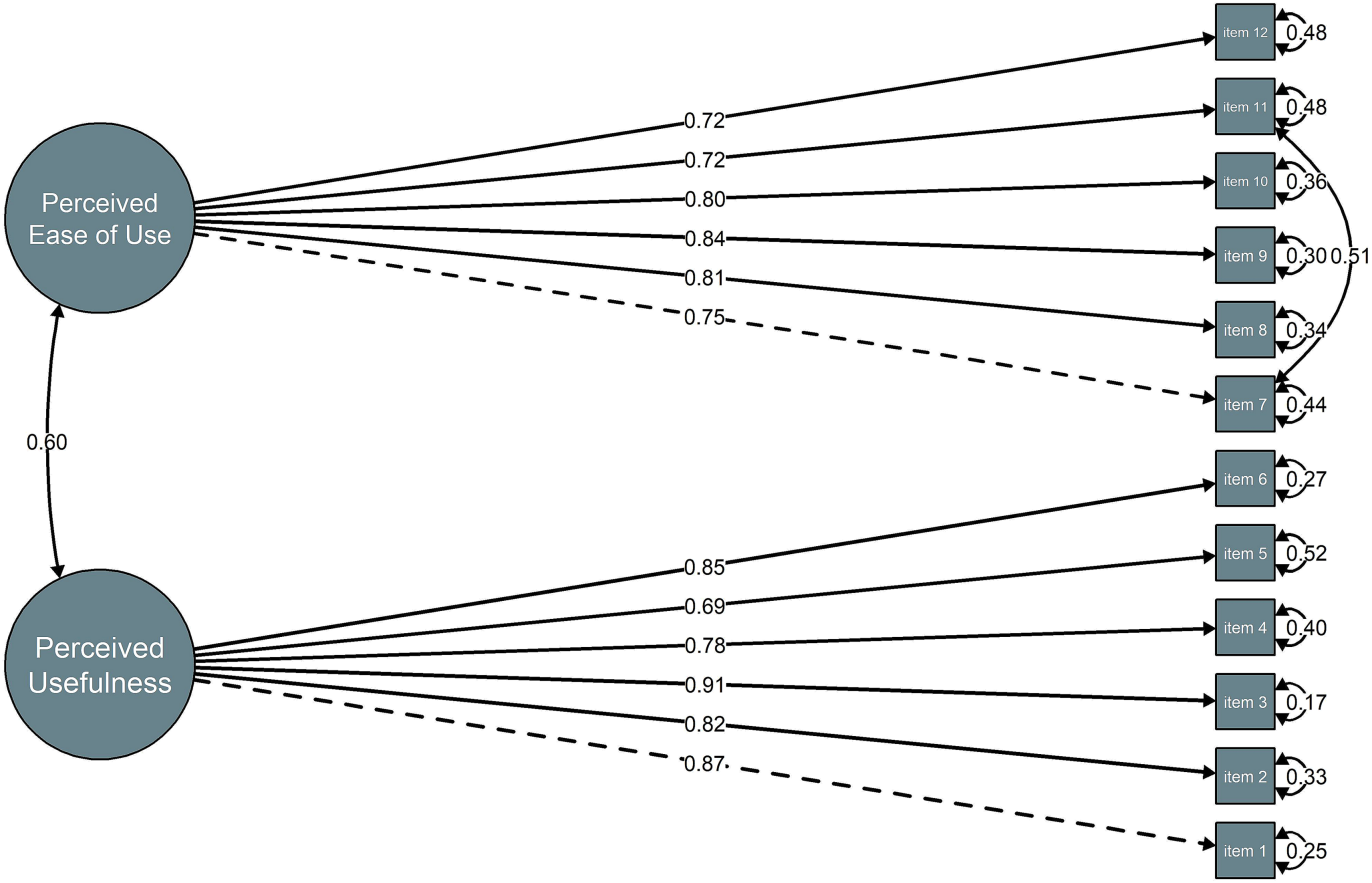
Factorial structure of TAM-12.

Confirmatory factor analysis supported the two-dimensional structure of TAM-12, showing good model fit (*X*^2^ = 198.36, gl = 52, *p* = 0.001, CFI = 0.92, RMSEA = 0.07 [90% CI: 0.067, 0.083], SRMR = 0.05), after correlating the error terms between items 7 and 11 (MI = 109.69). The only residual covariance freely estimated in the model was between items 7 and 11, which was statistically significant (Estimate = 0.142, SE = 0.050, *z* = 2.83, *p* = 0.005; standardized estimate = 0.505). No additional residual covariances were estimated, and all remaining error covariances were fixed to zero. Additionally, standardized factor loadings ranged from 0.69 to 0.91, indicating that all items contributed significantly to their respective dimensions (Figure 1).

### Reliability

The posterior estimation of the *ω* coefficient for the perceived usefulness dimension (ω = 0.92 [95% CI: 0.91, 0.93]) and for the perceived ease of use dimension (ω = 0.90 [95% CI: 0.88, 0.92]) was satisfactory. In addition, it showed adequate convergence, with Rhat = 1.000 for both dimensions. Likewise, the reliability coefficient H for the perceived usefulness dimension (H = 0.93) and for the perceived ease of use dimension (H = 0.90) were adequate, which reinforces the internal consistency of TAM-12.

### Evidence based on the relationship with other variables

**Figure 2:**
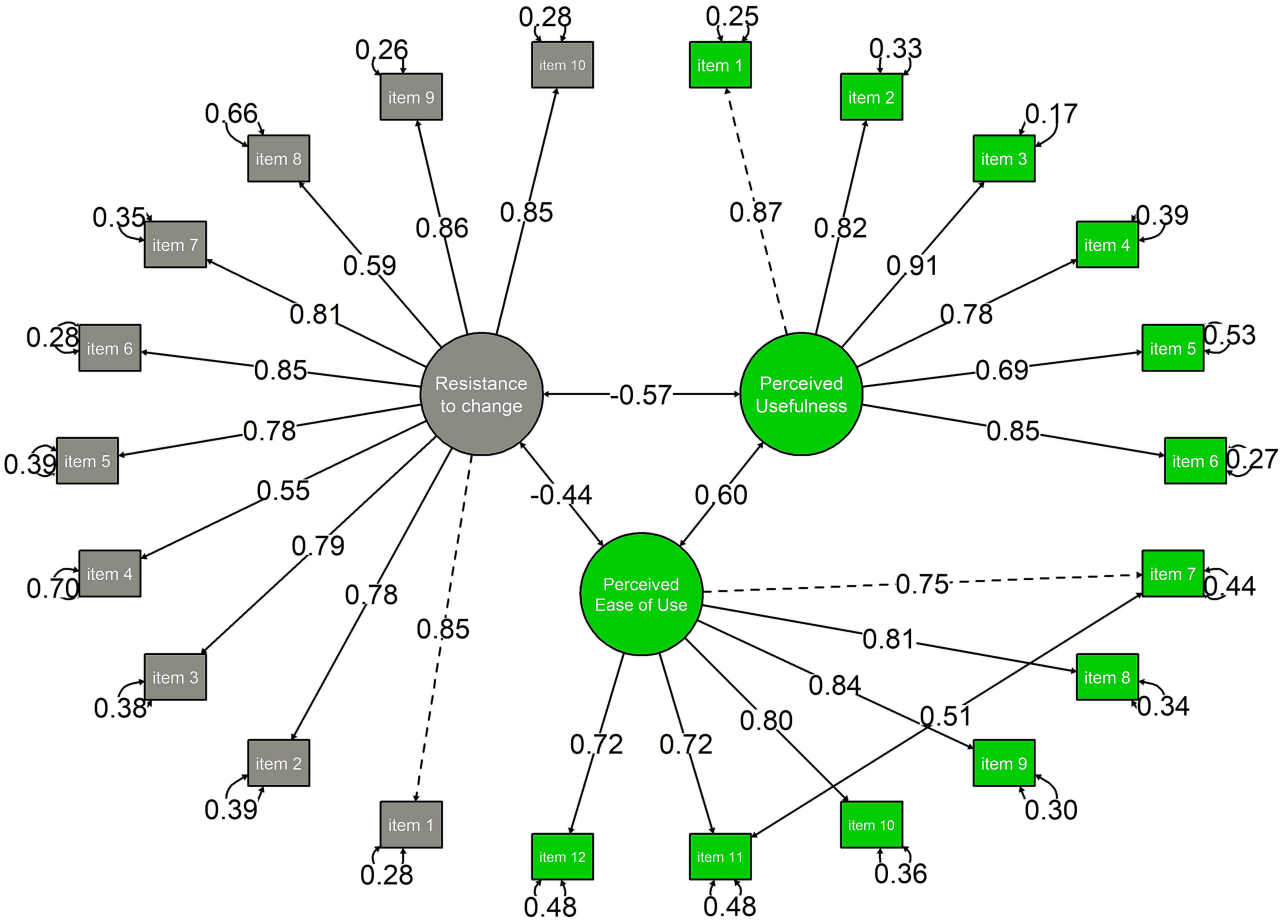
Structural model of the relationship between the dimensions of technology acceptance and resistance to change.

Figure 2 illustrates the results of the correlation analysis examining the associations between technology acceptance and resistance to change included in the model. The estimated model showed an adequate fit to the data: X^2^ = 819.16, gl = 205, *p* = 0.001, CFI = 0.90, RMSEA = 0.07 [90% CI: 0.073, 0.082], SRMR = 0.06, supporting the robustness of the proposed relational structure.

The results showed that the dimensions perceived usefulness and perceived ease of use were negatively and statistically significantly associated with resistance to change. Specifically, a large-magnitude association was observed between perceived usefulness and resistance to change (*r* = −0.57, IC 95% [−0.62, −0.51], *p* = 0.001), while a moderate-magnitude association was identified between perceived ease of use and resistance to change (*r* = −0.44, IC 95% [−0.51, −0.37], *p* = 0.001).

## Discussion

Technology acceptance in workplace environments has become an increasingly relevant phenomenon in the context of digital transformation, due to its close link with productivity, worker well-being, and organizational sustainability (Venkatesh et al., 2016). In this context, having psychometrically robust and culturally adapted instruments is essential to accurately measure workers’ attitudes and perceptions toward technologies (Sagnier et al., 2020). However, evidence regarding the validity and reliability of the Technology Acceptance Model (TAM) in Spanish-speaking populations is limited, as there is no validated Spanish adaptation, which restricts its measurement and research (Rafikasari et al., 2025). In response to this need, the present study aimed to translate TAM-12 into Spanish and analyze its psychometric properties in adult workers from the private sector in Lima, providing evidence through a structural equation modeling approach and a Bayesian reliability framework.

Regarding the internal structure, the results supported the two-dimensional model of TAM-12, composed of the dimensions of perceived usefulness and perceived ease of use, with fit indices reaching adequate levels after correlating the error terms between items 7 and 11. This is justifiable due to the similarity of their content, as both items relate to learning and skill in using the technological product (Davis, 1989). This structure is consistent with Davis’s (1989) original theoretical proposal and with previous psychometric studies conducted in different cultural and linguistic contexts (Grandón et al., 2021; Navarro et al., 2023; Silvestre et al., 2022). The replication of the bifactorial structure in a sample of Peruvian workers from the private technology sector reinforces the stability of the construct beyond the educational contexts in which it has been predominantly validated (Lobos et al., 2022; Pino Varela, 2022), and suggests that perceptions of usefulness and ease of use constitute distinguishable and empirically identifiable dimensions even in work environments with high technological exposure. From an applied perspective, this bifactorial structure facilitates differentiated interpretation of the instrument, allowing the identification of which of the two dimensions represents a greater barrier to technological adoption within a specific organization or work team—information that is valuable for the design of targeted organizational interventions (Yurder, 2025).

The present findings should be interpreted in continuity with previous TAM validation studies rather than as evidence of a new theoretical structure. Consistent with prior research conducted in Spanish-speaking and educational contexts, the two-factor model composed of perceived usefulness and perceived ease of use was retained. However, unlike previous studies that have predominantly focused on students, teachers, or general users, the present research examined TAM-12 in workers from the Peruvian private technology sector, a population exposed to continuous digital transformation and organizational change. Therefore, the main contribution of this study is not the proposal of a new model of technology acceptance, but the contextual verification of the instrument’s functioning in a Peruvian organizational setting.

Regarding reliability, the results showed adequate reliability for both dimensions. These values are consistent with previous literature reporting high internal consistency of TAM-12 across different cultural contexts (Navarro et al., 2023; Silvestre et al., 2022; Venkatesh and Davis, 2000), confirming that the instrument maintains its psychometric robustness in the studied sample. The use of a Bayesian approach represents a relevant methodological strength of this study, as it allows estimation of the uncertainty associated with reliability through credibility intervals, providing a more informative probabilistic interpretation than classical approaches based on the alpha coefficient (Pfadt et al., 2023). Unlike Cronbach’s alpha, which assumes tau-equivalence among items and is sensitive to sample size, Bayesian omega incorporates prior information about parameters and generates more stable estimates in moderate sample sizes (Pfadt et al., 2022). This type of estimation is especially useful in applied organizational research, where measurement precision has direct implications for decision-making regarding the selection and design of assessment tools (Baños-Chaparro and Caycho-Rodríguez, 2024). Additionally, coefficient H, which reflects factor replicability across samples, exceeded the threshold of 0.90 in both dimensions, indicating that the latent construct is well represented by its indicators and that the results would be stable in replication studies (Gunnell, 2020).

Regarding validity based on relationships with other variables, negative and statistically significant associations were found between both dimensions of TAM-12 and resistance to change. These findings are consistent with previous literature identifying resistance to change as a significant barrier to technological adoption (Green, 2024; Rahman et al., 2024; Seppänen et al., 2025). The stronger association observed between perceived usefulness and resistance to change can be explained by TAM’s theoretical framework: when a worker does not perceive that a technology will improve job performance, reluctance to adopt it intensifies, as the perception of instrumental benefit is one of the main determinants of adoption behavior (Davis, 1989; Marikyan et al., 2023). In contrast, the moderate relationship between perceived ease of use and resistance to change suggests that although perceived effort also influences opposition to technological change, its impact is lower than that of perceived usefulness in this specific context, which aligns with previous studies highlighting technological self-efficacy as a moderating factor in this relationship (Van der Westhuizen, 2021). Overall, these results provide evidence of validity based on relationships with other variables for TAM-12 and support its sensitivity in capturing relevant psychosocial dynamics in digitally transforming work environments.

Accordingly, the contribution of this study should be understood as incremental and contextual rather than as a theoretical extension or reformulation of TAM. The findings do not propose a new model of technology acceptance; rather, they provide initial psychometric evidence that the Spanish version of TAM-12 preserves its two-factor structure and adequate reliability in a Peruvian organizational sample. The added value of the Peruvian adaptation lies not only in translating the instrument into Spanish, but also in examining its semantic and cultural suitability, its internal structure, its reliability through Bayesian omega and coefficient H, and its theoretically consistent relationship with resistance to change. This is relevant because linguistic equivalence alone does not guarantee that an instrument will operate adequately in a specific occupational and cultural context (Davis, 1989; Venkatesh and Davis, 2000). From a practical perspective, TAM-12 may serve as a brief and useful tool for assessing technology acceptance among adult workers in the Peruvian private sector, particularly in organizational settings undergoing digital transformation (Cordero et al., 2023; Vélez and Morero, 2024). Its use could support the identification of workers or teams with lower willingness to adopt technology and inform training, technological support, and change management strategies (Inter-American Development Bank (IDB), 2023; United Nations Conference on Trade and Development, 2023). However, further studies are needed to expand its validity evidence across more diverse Peruvian and Latin American work settings.

Among the strengths of the study are the sample size, the Bayesian estimation of reliability, and the rigorous process of translation and cultural adaptation of the instrument, which included back-translation, expert judgment, and pilot testing with cognitive probing. These features address methodological limitations of previous studies and provide new psychometric evidence of TAM-12 in a previously unstudied context. However, some limitations should also be considered. First, due to the use of non-probability convenience sampling, the results are not generalizable to the entire workforce of the private technology sector in Metropolitan Lima; future research should employ probabilistic sampling strategies to improve representativeness and allow generalization of findings. Second, the cross-sectional design prevents evaluation of the instrument’s temporal stability; therefore, future studies should use test–retest or longitudinal designs to examine score consistency over time. Third, the sample consisted mainly of young adults with university education, which may introduce representativeness biases regarding workers with different educational backgrounds or older age; thus, more diverse samples are recommended in future research. Finally, other sources of validity, such as item response theory and measurement invariance across subgroups (e.g., sex, work modality, or years of experience). Although the sample showed relatively balanced distributions for sex and work modality, preliminary exploration of the invariance models revealed sparse or empty Likert response categories in some item-by-group combinations. This affected the stability of the estimation process and prevented the obtainment of reliable initial invariance models. Therefore, comparisons of TAM-12 scores across these groups should be interpreted with caution until future studies with larger, more heterogeneous, and adequately distributed samples can evaluate configural, metric, and scalar invariance. These pending analyses represent an important agenda for future research aimed at consolidating the psychometric evidence of the instrument in the Peruvian context.

The non-probability convenience sampling strategy and recruitment through social media may have overrepresented workers with greater digital access, higher familiarity with online surveys, stronger interest in technology, or more favorable attitudes toward technological innovation. Therefore, the findings should not be interpreted as representative of all workers in the Peruvian technology sector. Rather, they provide initial psychometric evidence for a specific group of adult workers from Metropolitan Lima who voluntarily participated in an online survey. This limitation restricts the external validity of the results and highlights the need for future studies using probabilistic, stratified, or company-based sampling frames that include workers from different regions, organizational sizes, positions, and levels of technological exposure.

## Conclusion

This study provides initial psychometric evidence regarding the Spanish version of TAM-12 in adult workers from the private technology sector in Metropolitan Lima, supporting its bifactorial structure, reliability, and validity based on relationships with other variables. These findings support the use of TAM-12 as a valid and reliable tool for assessing technology acceptance in this population, contributing to the advancement of research in organizational psychology and professional practice in digital transformation contexts. Overall, these results contribute to the available psychometric evidence on TAM-12 in Latin American organizational contexts, although they should be interpreted as an initial and context-specific contribution rather than as a theoretical extension of the model.

## Data Availability

The raw data supporting the conclusions of this article will be made available by the authors, without undue reservation.

## References

[ref1] Arifin W. N. (2026) Sample size calculator (web). Available online at: http://wnarifin.github.io (Accessed February 19, 2026).

[ref2] Ato M. López-García J. J. Benavente A. (2013). A classification system for research designs in psychology. Ann. Psychol. 29, 1038–1059. doi: 10.6018/analesps.29.3.178511

[ref4] Baños-Chaparro J. Caycho-Rodríguez T. (2024). Coeficiente omega bayesiano: aplicaciones en ciencias de la salud. Med. Clin. Soc. 8, 244–247. doi: 10.52379/mcs.v8i2.401

[ref5] Ciarli T. Kenney M. Massini S. Piscitello L. (2021). Digital technologies, innovation, and skills: emerging trajectories and challenges. Res. Policy 50:104289. doi: 10.1016/j.respol.2021.104289

[ref6] Cirillo V. Fanti L. Mina A. Ricci A. (2023). The adoption of digital technologies: investment, skills, work organisation. SSRN Electron. J. doi: 10.2139/ssrn.4181459

[ref7] Cohen J. (1988). Statistical power Analysis for the Behavioral Sciences. 2nd Edn Abingdon: Routledge.

[ref8] Colegio de Psicólogos del Perú. Código de ética y deontología Lima Colegio de Psicólogos del Perú (2024) Available online at: https://www.cpsp.pe/images/documentos/marco_legal/CPsP_CDN_codigo_de_etica_y_deontologia.pdf (Accessed February 20, 2026).

[ref9] Cordero D. Altamirano K. L. Parra J. O. Espinoza W. S. (2023). Intention to adopt industry 4.0 by organizations in Colombia, Ecuador, Mexico, Panama, and Peru. IEEE Access 11, 8362–8386. doi: 10.1109/ACCESS.2023.3238384

[ref10] Davis F. D. (1989). Perceived usefulness, perceived ease of use, and user acceptance of information technology. MIS Q. 13, 319–339. doi: 10.2307/249008

[ref11] De la Peña-López I. J. Acosta-Gonzaga E. (2025). Adoption of technology in older adults in Mexico City: an approach from the technology acceptance model. Brain Sci. 15:632. doi: 10.3390/brainsci15060632, 40563802 PMC12191114

[ref12] DeLange Martinez P. Tancredi D. Pavel M. Garcia L. Young H. M. (2025). Adapting the technology acceptance model to examine the use of information communication technologies and loneliness among low-income, older Asian Americans: cross-sectional survey analysis. JMIR Aging 8:e63856. doi: 10.2196/63856, 39778204 PMC11754985

[ref13] Espina-Romero L. Parra D. Hurtado H. Rodriguez E. Arias-Montoya F. Noroño-Sánchez J. . (2024). The role of digital transformation and digital competencies in organizational sustainability: a study of SMEs in Lima, Peru. Sustainability 16:6993. doi: 10.3390/su16166993

[ref14] Gosling S. Mason W. (2015). Internet research in psychology. Annu. Rev. Psychol. 66, 877–902. doi: 10.1146/annurev-psych-010814-015321 25251483

[ref15] Grandón E. E. Díaz-Pinzón B. Magal S. R. Rojas-Contreras K. (2021). Technology acceptance model validation in an educational context: a longitudinal study of ERP system use. J. Inf. Syst. Eng. Manag. 6:em0134. doi: 10.29333/jisem/9582

[ref16] Green G. (2024). Analysis of the mediating effect of resistance to change, perceived ease of use, and behavioral intention to use technology-based learning among younger and older nursing students. J. Prof. Nurs. 50, 66–72. doi: 10.1016/j.profnurs.2023.11.003, 38369374

[ref17] Gunnell K. E. (2020). “Validity and reliability,” in The Routledge International Encyclopedia of Sport and Exercise Psychology, eds. Hackfort D. Schinke R. (Abingdon: Routledge), 632–645.

[ref18] Hilbig B. E. Thielmann I. Böhm R. (2022). Bending our ethics code: avoidable deception and its justification in psychological research. Eur. Psychol. 27, 62–70. doi: 10.1027/1016-9040/a000431

[ref19] Hilda A. G. Morán R. O. Lizana N. P. Pedro M. S. Yasser S. L. Llonto Y. . (2024). Digital transformation and its relationship to the job performance of employees at a private university in Peru. F1000Res 13:692. doi: 10.12688/f1000research.151251.1 39323977 PMC11422760

[ref3] Inter-American Development Bank (IDB). (2023) Peru to Advance its digital Transformation with IDB Support Available online at: https://www.iadb.org/en/news/peru-advance-its-digital-transformation-idb-support (Accessed February 20, 2026).

[ref20] Jordan Muiños F. M. (2021). Valor de corte de los índices de ajuste en el análisis factorial confirmatorio. Psocial, 7(1), 1–5. https://publicaciones.sociales.uba.ar/index.php/psicologiasocial/article/view/6764/579

[ref21] Kline R. B. (2023). Principles and Practice of Structural Equation Modeling. Fifth Edn New York: The Guilford Press.

[ref22] Knapp S. VandeCreek L. (2003). An overview of the major changes in the 2002 APA ethics code. Prof. Psychol. Res. Pract. 34, 301–308. doi: 10.1037/0735-7028.34.3.301, 16471011

[ref23] Lobos K. Cobo-Rendón R. C. Guzmán E. Bruna C. (2022). Adaptación y validación de dos cuestionarios sobre implementación de la tecnología en la docencia universitaria. Form. Univ. 15, 1–14. doi: 10.4067/S0718-50062022000500001

[ref24] Luna-Victoria A. Guevara C. (2021) Adaptación y propiedades psicométricas de la escala resistance to change (RTC-11) (Doctoral dissertation, Tesis de Licenciatura, Universidad de Lima]. Repositorio de la Universidad de Lima. Available online at: https://repositorio.ulima.edu.pe/handle/20.500.12724/14796 (Accessed February 20, 2026).

[ref25] Marikyan D. Papagiannidis S. Stewart G. (2023). Technology acceptance research: Meta-analysis. J. Inf. Sci. 52, 429–450. doi: 10.1177/01655515231191177, 42479872

[ref26] Navarro R. Baldeon G. García A. Bernal V. (2023). Adaptación del modelo de aceptación de tecnologías para explorar las intenciones de uso en la educación virtual. Digit. Educ. Rev. 44, 13–22. doi: 10.1344/der.2023.44.13-22

[ref27] Oreg S. (2003). Resistance to change: developing an individual differences measure. J. Appl. Psychol. 88, 680–693. doi: 10.1037/0021-9010.88.4.680, 12940408

[ref28] Pfadt J. M. van den Bergh D. Moshagen M. (2023). Classical and Bayesian uncertainty intervals for the reliability of multidimensional scales. Struct. Equ. Model. 30, 349–363. doi: 10.1080/10705511.2022.2124162

[ref29] Pfadt J. M. van den Bergh D. Sijtsma K. Moshagen M. Wagenmakers E. J. (2022). Bayesian estimation of single-test reliability coefficients. Multivar. Behav. Res. 57, 620–641. doi: 10.1080/00273171.2021.1891855, 33759671

[ref30] Pino Varela J. (2022). Validación del Modelo de Aceptación Tecnológica (TAM) para medir la competencia digital en estudiantes de Educación Primaria. EDMETIC 11:6. doi: 10.21071/edmetic.v11i1.13508

[ref31] Rafikasari E. F. Iriawan N. Otok B. W. (2025). Bayesian restructuring technology acceptance model (TAM) with moderating lecture self-managing in e-learning adoption. Decis. Mak.: Appl. Manag. Eng 8, 517–533. doi: 10.31181/dmame8120251427

[ref32] Rahman M. H. Ahmad A. B. Mohamed Sawal M. Z. H. B. (2024). The influence of personal factors on resistance to technology adoption in university libraries in Bangladesh. Inf. Dev. doi: 10.1177/02666669241257196 (online ahead of print).

[ref33] Roebianto A. Savitri S. I. Aulia I. Suciyana A. Mubarokah L. (2023). Content validity: definition and procedure of content validation in psychological research. Test. Psychomet. Methodol. Appl. Psychol. 30, 5–18. doi: 10.4473/TPM30.1.1

[ref34] Sagnier C. Loup-Escande E. Lourdeaux D. Thouvenin I. Valléry G. (2020). User acceptance of virtual reality: an extended technology acceptance model. Int. J. Hum.-Comput. Interact. 36, 993–1007. doi: 10.1080/10447318.2019.1708612

[ref35] Scholkmann A. B. (2021). “Resistance to (digital) change: individual, systemic and learning-related perspectives,” in Digital Transformation of Learning Organizations, (Berlin: Springer), 219–236.

[ref36] Seppänen S. Ukko J. Saunila M. (2025). Understanding determinants of digital transformation and digitizing management functions in incumbent SMEs. Digital Business 5:100106. doi: 10.1016/j.digbus.2025.100106

[ref37] Silvestre E. Miranda A. M. Gutiérrez V. F. (2022). Validation of a technology acceptance model (TAM) in Dominican university students. Educación 31, 113–136. doi: 10.18800/educacion.202201.005

[ref38] United Nations Conference on Trade and Development (2023) UNCTAD Assessment set to Boost digital Economy in Peru. Available online at: https://unctad.org/news/unctad-assessment-set-boost-digital-economy-peru (Accessed February 20, 2026).

[ref39] Van der Westhuizen R. (2021) Effect of Emotional Intelligence on Resistance towards 4IR Technology Changes [Tesis de maestría, University of Johannesburg]. UJContent Repository. Available online at: https://hdl.handle.net/10210/485608 (Accessed February 21, 2026).

[ref40] Vélez J. G. Morero H. (2024). Los desafíos de la digitalización en Pymes de América Latina: un recorrido sobre los avances empíricos recientes. Pymes, Innovación y Desarrollo. Available online at: https://ri.conicet.gov.ar/handle/11336/238009. pp. 1–25

[ref41] Venkatesh V. Davis F. D. (2000). A theoretical extension of the technology acceptance model: four longitudinal field studies. Manag. Sci. 46, 186–204. doi: 10.1287/mnsc.46.2.186.11926, 19642375

[ref42] Venkatesh V. Thong J. Y. Xu X. (2016). Unified theory of acceptance and use of technology: a synthesis and the road ahead. J. Assoc. Inf. Syst. 17, 328–376. doi: 10.17705/1jais.00428

[ref43] Yurder Y. (2025). Revisiting the technology acceptance model in the age of artificial intelligence: a bibliometric analysis of TAM in business and marketing literature. J. Res. Bus. 10, 497–517. doi: 10.54452/jrb.1615746

